# ﻿Three new species of the *Clubionacorticalis* group (Araneae, Clubionidae) from China

**DOI:** 10.3897/zookeys.1157.99674

**Published:** 2023-04-05

**Authors:** Panlong Wu, Yang Chen, Feng Zhang

**Affiliations:** 1 Collaborative Innovation Center for Grassland Ecological Security, Ministry of Education of China, School of Ecology and Environment, Inner Mongolia University, Hohhot 010021, China Hebei University Hebei China; 2 The Key Laboratory of Zoological Systematics and Application, Institute of Life Science and Green Development, College of Life Sciences, Hebei University, Baoding, Hebei 071002, China Inner Mongolia University Hohhot China; 3 Shanxi Yuncheng Vocational and Technical College of Agricultural, Yuncheng, Shanxi 044000, China Shanxi Yuncheng Vocational and Technical College of Agricultural Shanxi China

**Keywords:** Clubionids, sac spiders, species diversity, taxonomy

## Abstract

Three new species of the *Clubionacorticalis* group in China are described: *Clubionabidactylina***sp. nov.**, *C.camela***sp. nov.**, and *C.subhuiming***sp. nov.**

## ﻿Introduction

*Clubiona* Latreille, 1804, the largest genus in the family Clubionidae, currently includes 519 species distributed on most continents of the world (WSC 2023). Due to its high species diversity and worldwide distribution, several arachnologists have proposed to subdivide this genus into subgenera and species groups (e.g., Simon 1932; Lohmander 1944; Edwards 1958; Wiehle 1965; Dondale and Redner 1982; Mikhailov 1995, 2012; Deeleman-Reinhold 2001; Wunderlich 2011).

The *Clubionacorticalis* group, first recognised by Simon (1932), has recently been one of the most frequently reported species groups of the family (Marusik and Omelko 2018; Zhang et al. 2021; Zhong et al. 2022). In China, at least 51 species of the *Clubionacorticalis* group have been recorded to date, and these are mainly distributed in south China (Table 1). While examining Chinese clubionids, we found three new species belonging to the *Clubionacorticalis* group, and we describe them in this paper.

**Table 1.** Species belonging to the *Clubionacorticalis* group recorded in China.

	Species name	Known sex	Distribution
1	*C.pyrifera* Schenkel, 1936	♂♀	Gansu
2	*C.kurosawai* Ono, 1986	♂♀	Taiwan
3	*C.parallela* Hu & Li, 1987	♂♀	Tibet
4	*C.yaginumai* Hayashi, 1989	♂♀	Taiwan
5	*C.lyriformis* Song & Zhu, 1991	♀	Hubei
6	*C.moralis* Song & Zhu, 1991	♂♀	Hubei
7	*C.taiwanica* Ono, 1994	♂♀	Yunnan, Taiwan
8	*C.didentata* Zhang & Yin, 1998	♂♀	Yunnan
9	*C.qiyunensis* Xu, Yang & Song, 2003	♂♀	Fujian, Anhui
10	*C.altissimoides* Liu et al., 2007	♂♀	Yunnan
11	*C.applanata* Liu et al., 2007	♂♀	Yunnan
12	*C.cylindrata* Liu et al., 2007	♂♀	Yunnan
13	*C.lamina* Zhang, Zhu & Song, 2007	♂	Yunnan
14	*C.tengchong* Zhang, Zhu & Song, 2007	♂	Yunnan
15	*C.cordata* Zhang & Zhu, 2009	♂♀	Sichuan, Tibet
16	*C.kai* Jäger & Dankittipakul, 2010	♂♀	Yunnan
17	*C.submoralis* Wu, Zheng & Zhang, 2015	♂♀	Yunnan
18	*C.pollicaris* Wu, Zheng & Zhang, 2015	♂♀	Yunnan
19	*C.pianmaensis* Wang, Wu & Zhang, 2015	♂♀	Yunnan
20	*C.cochleata* Wang, Wu & Zhang, 2015	♂♀	Sichuan
21	*C.biforamina* Liu, Peng & Yan, 2016	♂♀	Yunnan
22	*C.dactylina* Liu, Peng & Yan, 2016	♂♀	Yunnan
23	*C.falciforma* Liu, Peng & Yan, 2016	♂♀	Yunnan
24	*C.gongshan* He, Liu & Zhang, 2016	♂♀	Yunnan
25	*C.lucida* He, Liu & Zhang, 2016	♂♀	Hunan
26	*C.multidentata* Liu, Peng & Yan, 2016	♂♀	Yunnan
27	*C.tangi* Liu, Peng & Yan, 2016	♂♀	Yunnan
28	*C.bifurcata* Zhang, Yu & Zhong, 2018	♂♀	Guizhou
29	*C.dichotoma* Wang, Chen & Zhang, 2018	♂♀	Guizhou
30	*C.globosa* Wang, Chen & Zhang, 2018	♂♀	Guizhou
31	*C.lamellaris* Zhang, Yu & Zhong, 2018	♂♀	Guizhou
32	*C.subapplanata* Wang, Chen & Zhang, 2018	♂♀	Guizhou
33	*C.fanjingshan* Wang, Chen & Zhang, 2018	♂	Guizhou
34	*C.huiming* Wang, Chen & Zhang, 2018	♂	Guizhou
35	*C.subcylindrata* Wang, Chen & Zhang, 2018	♂	Guizhou
36	*C.cochlearis* Yu & Li, 2019	♂♀	Yunnan
37	*C.tiane* Yu & Li, 2019	♂♀	Yunnan
38	*C.subrama* Yu & Li, 2019	♂♀	Yunnan
39	*C.subyaginumai* Yu & Li, 2019	♂♀	Yunnan
40	*C.caohai* Zhang & Yu, 2020	♂♀	Guizhou
41	*C.dakong* Zhang & Yu, 2020	♀	Tibet
42	*C.yanzhii* Zhang & Yu, 2020	♀	Hunan
43	*C.dengpao* Yu & Li, 2021	♀	Yunnan
44	*C.subdidentata* Yu & Li, 2021	♀	Yunnan
45	*C.tixing* Yu & Li, 2021	♀	Yunnan
46	*C.xiaoci* Yu & Li, 2021	♂♀	Yunnan
47	*C.xiaokong* Yu & Li, 2021	♀	Yunnan
48	*C.yejiei* Yu & Li, 2021	♀	Yunnan
49	*C.zhaoi* Yu & Li, 2021	♀	Yunnan
50	*C.zhigangi* Yu & Li, 2021	♂♀	Yunnan
51	*C.xianning* Zhong & Yu, 2022	♂♀	Hubei
52	*C.bidactylina* sp. nov.	♂♀	Tibet
53	*C.camela* sp. nov.	♂♀	Guangxi
54	*C.subhuiming* sp. nov.	♂♀	Hunan

## ﻿Materials and methods

All specimens studied are stored in 75% ethanol and stored in the Museum of Hebei University (**MHBU**), Baoding, China. We identified these specimens using a Tech XTL-II stereomicroscope, and drew, photographed, and measured them using a Leica M205A stereomicroscope equipped with a drawing tube and a DFC450 CCD camera. Carapace length was measured from the anterior margin to the posterior margin of the carapace medially. Eye sizes were measured as the maximum diameter of the lens in dorsal or frontal view. The leg measurements are shown as total length (femur, patella, tibia, metatarsus, tarsus). The epigyne was cleared in a warm 10% potassium hydroxide (KOH) solution and transferred to 75% ethanol for drawing, photographing, and measuring. All measurements are in millimetres.

The following abbreviations are used: **ALE**, anterior lateral eye; **AME**, anterior median eye; **B**, bursa; **C**, conductor; **CD**, copulatory duct; **CO**, copulatory opening; **E**, embolus; **FD**, fertilisation duct; **LPA**, lateral patellar apophysis; **MOA**, median ocular area; **PLE**, posterior lateral eye; **PME**, posterior median eye; **RFA**, retrolateral femoral apophysis; **RPA**, retrolateral patellar apophysis; **RTA**, retrolateral tibial apophysis; **S**, spermatheca; **SD**, sperm duct; **TA**, tegular apophysis; **VFA**, ventral femoral apophysis; **VPA**, ventral patellar apophysis; **VTA**, ventral tibial apophysis.

## ﻿Taxonomy

### ﻿ Clubiona bidactylina sp. nov.

Taxon classificationAnimaliaAraneaeClubionidae

28A47269-25E8-5C31-9142-BE53C6EADB77

https://zoobank.org/3D8E3EC9-2306-4EA9-929F-2B4DCF38743D

Figs 1 , 2

#### Type material.

***Holotype***: China • ♂; Tibet, Zayu County (28°39.59'N, 97°27.96'E, 2323 m elev.), 25 June 2018, Yannan Mu leg. ***Paratypes***: • 2♀, same data as the holotype.

#### Etymology.

The specific name is the combination of the *bi*- (two) and *dactylina* (finger-shaped), referring to two finger-shaped apophyses on the femur; adjective.

#### Diagnosis.

The new species resembles *C.pianmaensis* Wang, Wu & Zhang, 2015 (Wang et al. 2015: figs 1–14), but it differs by the presence of femoral ventral apophysis (vs. absent), 2 retrolateral tibial apophyses (vs. 1), the wider embolic base (embolic base width/genital bulb width ratio 0.4 vs. 0.2), conductor extended to the middle part of embolus (vs. tip of embolus), the smaller copulatory openings (copulatory opening width/interdistance ratio 0.2 vs. 4.7), and the boot-shaped bursae (vs. almost spherical).

#### Description.

**Figure 1.**
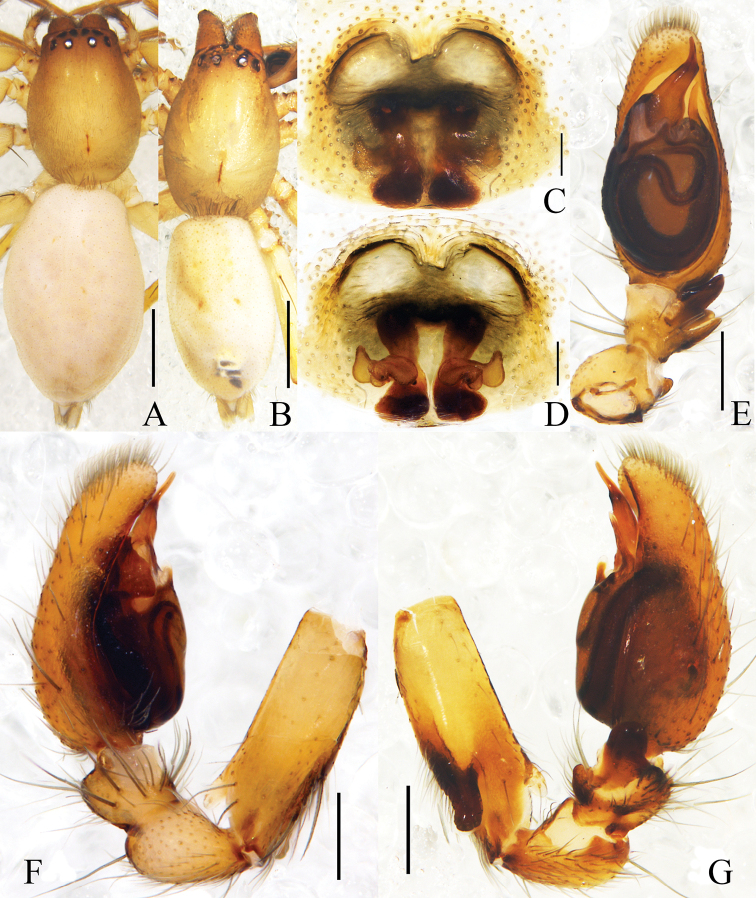
*Clubionabidactylina* sp. nov. **A** female habitus **B** male habitus **C** epigyne, ventral view **D** vulva, dorsal view. Left male palp: **E** ventral view **F** prolateral view **G** retrolateral view. Scale bars: 1 mm (**A, B**); 0.1 mm (**C, D**); 0.2 mm (**E–G**).

**Male** (Fig. 1B). Total length 4.11: carapace 2.04 long, 1.44 wide; abdomen 2.16 long, 1.31 wide. Carapace yellowish brown. Eye sizes and interdistances: AME 0.08, ALE 0.11, PME 0.10, PLE 0.12; AME–AME 0.09, AME–ALE 0.04, ALE–ALE 0.34, PME–PME 0.22, PME–PLE 0.12, PLE–PLE 0.66, ALE–PLE 0.11. MOA 0.23 long, front width 0.27, back width 0.42. Clypeus height 0.05. Chelicerae yellow brown, promargin with 4 teeth, retromargin with 3 teeth. Endites paler than chelicerae. Labium yellow-brown, 0.31 long, 0.24 wide. Sternum 1.07 long, 0.62 wide. Abdomen oval, light yellow, with conspicuous anterior tufts of setae. Legs, yellow, except tarsi and metatarsi yellowish brown, both tibia I and II with two pairs of ventral spines, both metatarsi I and II with 1 pair of ventral spines. Leg measurements: I 4.25 (1.25, 0.59, 1.09, 0.84, 0.48), II 5.01 (1.46, 0.67, 1.35, 1.00, 0.53), III 3.87 (1.15, 0.58, 0.79, 0.97, 0.38), IV 5.58 (1.62, 0.63, 1.29, 1.55, 0.49). Leg formula: 4-2-1-3.

**Figure 2.**
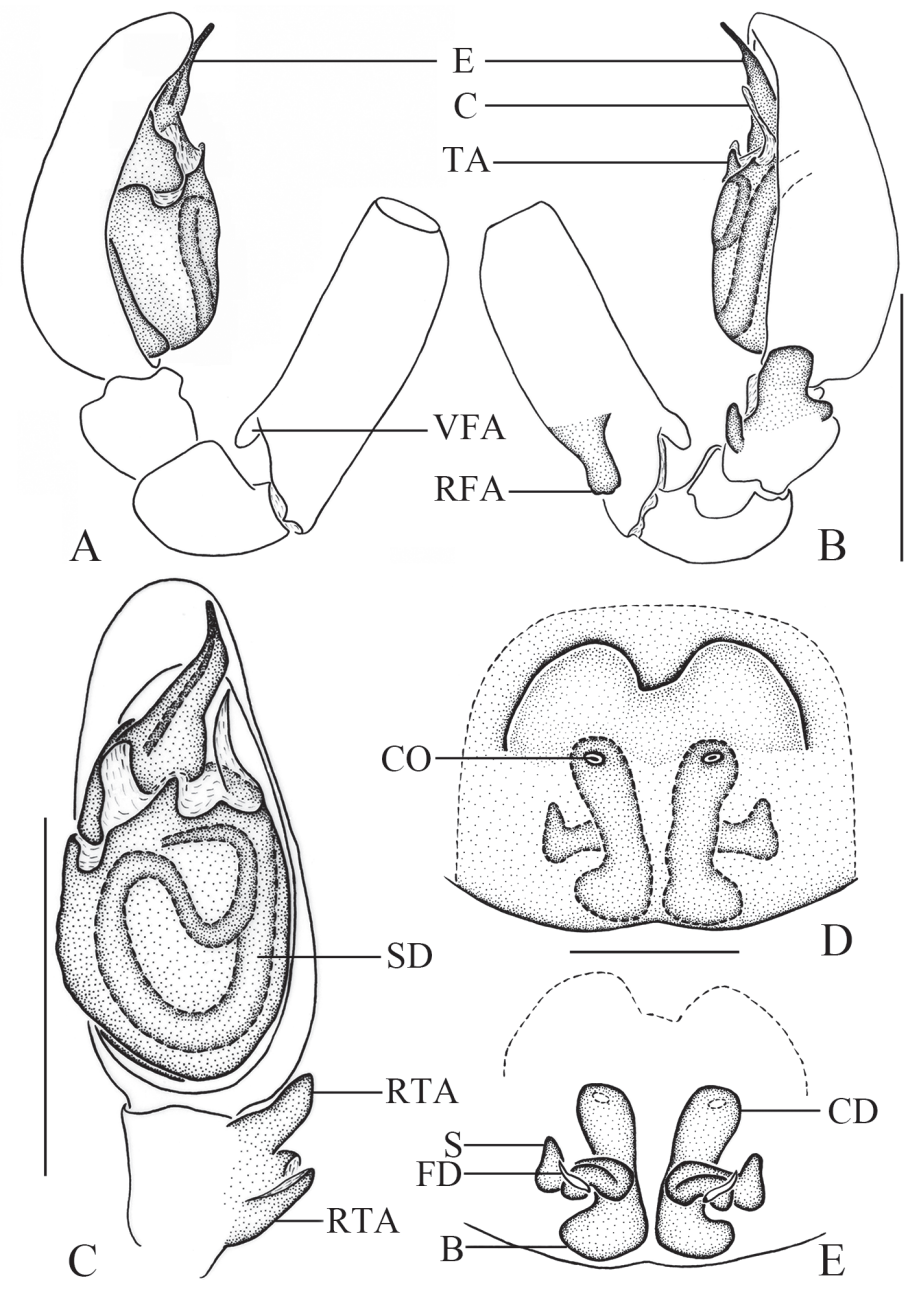
*Clubionabidactylina* sp. nov. Left male palp: **A** prolateral view **B** retrolateral view **C** ventral view **D** epigyne, ventral view **E** vulva, dorsal view. Scale bars: 0.5 mm (**A–C**); 0.1 mm (**D, E**).

Palp (Figs 1E–G, 2A–C). Femur length/width ratio 2.9, modified, with 2 apophyses, retrolateral apophysis digitiform, located medially, ventral apophysis transparent, located distally. Patella length/width ratio 1.5, unmodified. Tibia as long as wide, with 2 retrolateral apophyses, wedge-shaped apophysis located distally, with a small, triangular process at base in retrolateral view, digitiform apophysis located proximally. Cymbium length/femur length ratio 1.2. Cymbium length/width ratio 2.3, oblong. Tegulum uninflated, sperm a-shaped in ventral view. Conductor lance-shaped in ventral view, extended to the middle part of embolus. Embolus with wide base, embolic base width/genital bulb width ratio 0.4, the upper third sharply pointed and extended towards tip of cymbium.

**Female** (Fig. 1A). Total length 4.71–4.79; body length 4.79: carapace 2.04 long, 1.48 wide; abdomen 2.79 long, 1.83 wide. Eye sizes and interdistances: AME 0.08, ALE 0.11, PME 0.09, PLE 0.11; AME–AME 0.11, AME–ALE 0.04, ALE–ALE 0.36, PME–PME 0.23, PME–PLE 0.15, PLE–PLE 0.71, ALE–PLE 0.10. MOA 0.23 long, front width 0.27, back width 0.43. Clypeus height 0.05. Labium 0.30 long, 0.26 wide. Sternum 1.11 long, 0.74 wide. Leg measurements: I 3.52 (1.05, 0.56, 0.82, 0.66, 0.43), II 3.92 (1.18, 0.63, 0.92, 0.76, 0.43), III 3.35 (1.02, 0.44, 0.72, 0.81, 0.36), IV 5.19 (1.49, 0.72, 1.16, 1.40, 0.42). Leg formula: 4-2-1-3. Other characters as in male.

Epigyne (Figs 1C, D, 2D, E). Epigynal plate length/width ratio 0.9, the anterior part membranous, with 2 semicircular hoods. Copulatory openings small and circular, located medially, width/interdistance ratio 0.2. Copulatory ducts almost straight, descended obliquely, then connected with boot-shaped bursae. Bursae situated posteriorly, length/width ratio 1.6. Spermathecae tubular, almost located at the middle position between copulatory openings and bursae. Fertilisation ducts short and lance-shaped, located on dorsal-lateral surface of spermathecae.

#### Distribution.

Known only from the type locality.

### ﻿ Clubiona camela sp. nov.

Taxon classificationAnimaliaAraneaeClubionidae

5D9EE146-A844-57D2-89DE-21634E66BCC5

https://zoobank.org/1BF9FAAE-71AD-41DA-B59C-8D38521FFB89

Figs 3 , 4 , 5

#### Type material.

***Holotype***: China • ♂; Yunnan Province, Pingbian County, Daweishan National Forest Park (22°59'16''N, 103°57'01''E, 2124 m elev.), 28 October 2016, Guiqiang Huang leg. ***Paratypes***: • 3♀2♂, same data as holotype.

#### Etymology.

The species name is derived from the Latin *camela* (camel), referring to hump-shaped retrolateral tibia apophyses in retrolateral view; noun.

#### Diagnosis.

The new species resembles *C.biforamina* Liu, Peng & Yan, 2016 (Liu et al. 2016: figs 1–12), but it differs by the larger embolus length/conductor length ratio (1.6 vs. 0.6), triangle distal retrolateral tibia apophyses (vs. trapezoidal), the copulatory openings located almost centrally (vs. anteriorly), the coiled copulatory ducts (vs. U-shaped), the pear-shaped bursae (vs. oblong).

**Figure 3.**
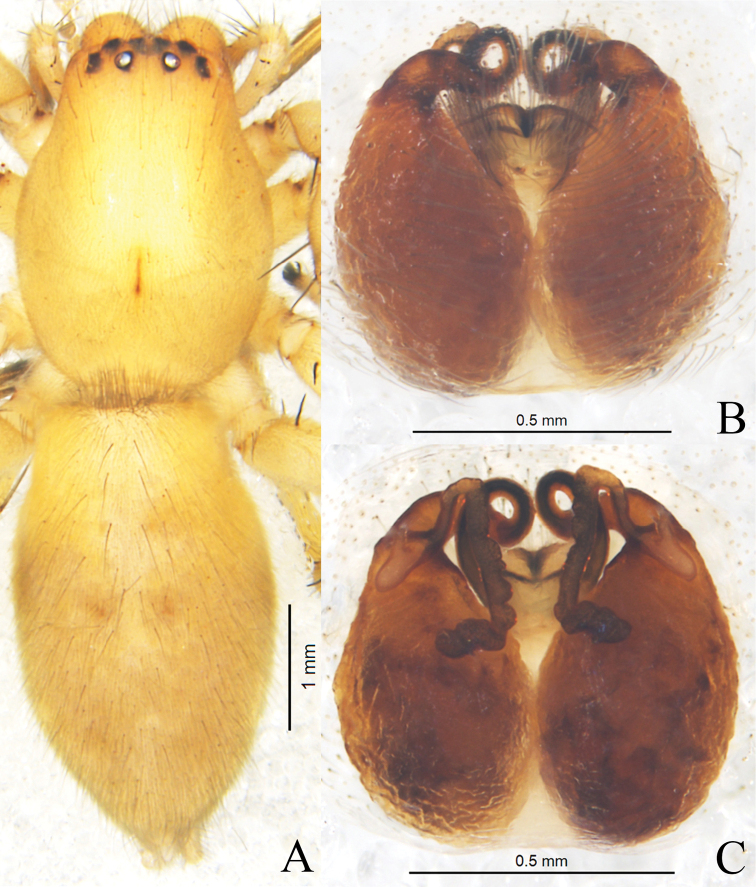
*Clubionacamela* sp. nov. **A** female habitus **B** epigyne, ventral view **C** vulva, dorsal view. Scale bars: 1 mm (**A**); 0.5 mm (**B, C**).

#### Description.

**Figure 4.**
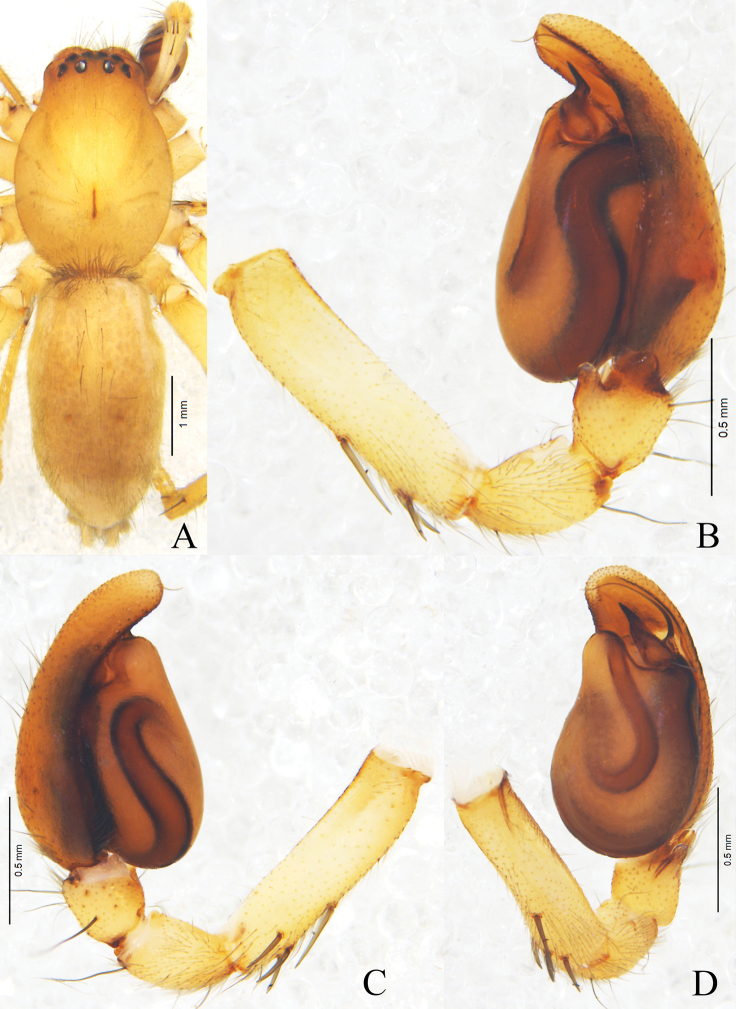
*Clubionacamela* sp. nov. **A** male habitus. Left male palp: **B** retrolateral view **C** prolateral view **D** ventral view. Scale bars: 1 mm (**A**); 0.5 mm (**B–D**).

**Male** (Fig. 4A). Holotype total length 6.21. Carapace 2.80 long, 1.99 wide; abdomen 3.51 long, 1.70 wide. Carapace yellowish brown. In dorsal view, anterior eye row recurved, posterior eye row almost straight. Eye sizes and interdistances: AME 0.12, ALE 0.15, PME 0.12, PLE 0.14; AME–AME 0.11, AME–ALE 0.06, PME–PME 0.25, PME–PLE 0.17, ALE–PLE 0.08. MOA 0.38 long, front width 0.34, back width 0.50. Clypeus height 0.09. Chelicerae yellowish, promargin with 5 teeth, retromargin with 6 teeth. Labium 0.52 long, 0.36 wide. Sternum 1.46 long, 1.03 wide. Endites yellow, longer than wide. Abdomen oval, brownish yellow, with conspicuous anterior tufts of setae; dorsum of abdomen with fine, yellow hairs; cardiac pattern brown. Spinnerets and legs yellow brown. Leg measurements: I 8.58 (2.40, 1.05, 2.43, 1.81, 0.89), II 12.31 (3.48, 1.20, 3.56, 2.83, 1.24), III 8.10 (2.67, 0.89, 1.80, 2.05, 0.69), IV 10.14 (2.92, 0.93, 2.42, 2.98, 0.89). Leg formula: 2-4-1-3.

**Figure 5.**
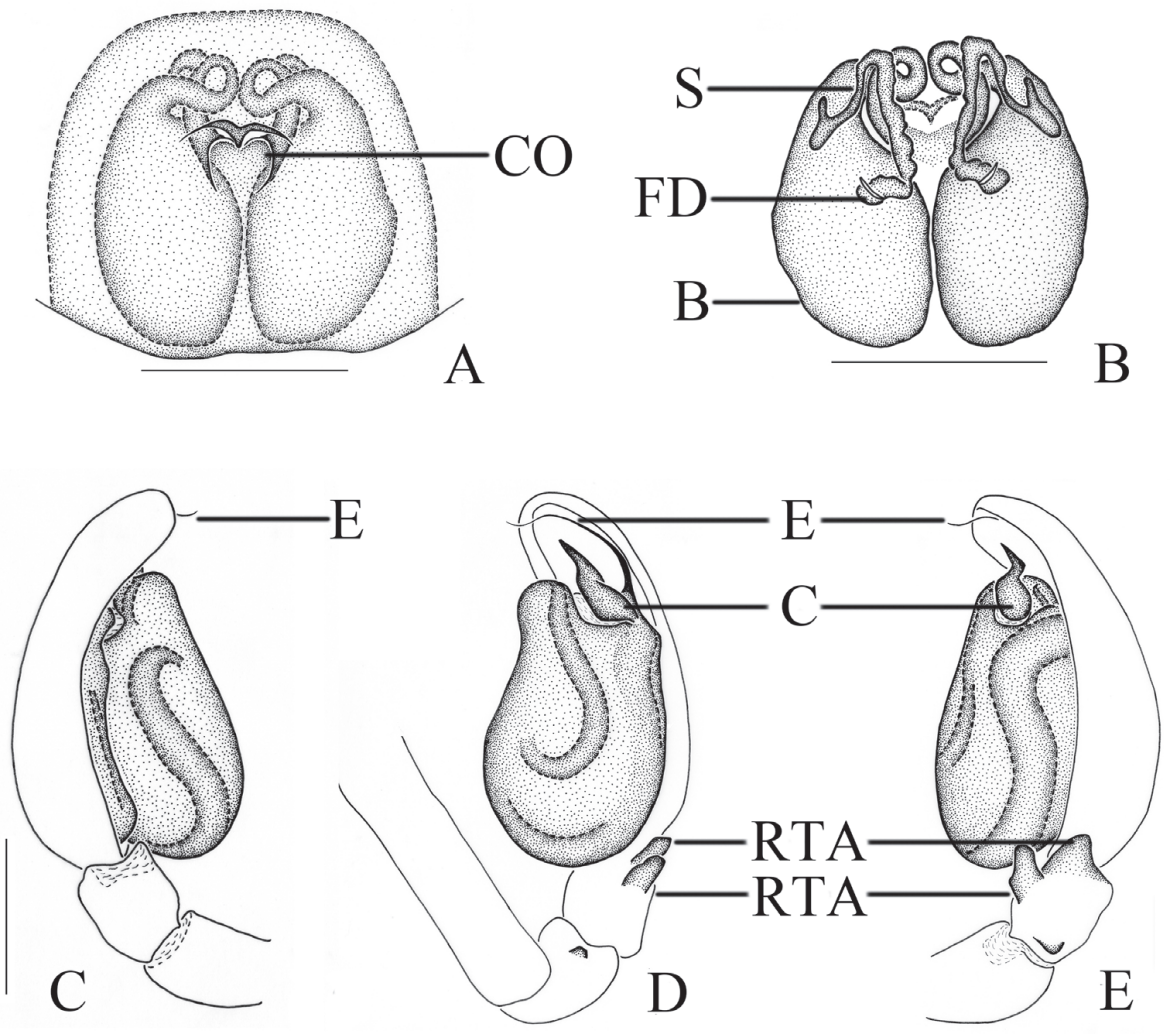
*Clubionacamela* sp. nov. **A** epigyne, ventral view **B** vulva, dorsal view. Left male palp: **C** prolateral view **D** ventral view **E** retrolateral view. Scale bars: 0.5 mm (**A–E**).

Palp (Figs 4B–D, 5C–E). Patella length/width ratio 1.7, with a small ventral apophysis. Patella length/tibia length 1.4. Tibia length/width ratio 1.1, with 2 thumb-shaped RTA in ventral view, hump-like in retrolateral view. Cymbium length/width ratio 3.1. Conductor length/width ratio 2.4, with oval base and beak-shaped distal part in retrolateral view, arose from retrolateral tegulum, then extended prolaterally. Embolus slender, rising from retrolatero-distal tegulum, gradually tapering, then extending to prolateral part of cymbium. Sperm duct obvious, S-shaped in prolateral view.

**Female** (Fig. 3A). Total length 6.12–6.51. One paratype measured: total length 6.51, carapace 2.77 long, 2.06 wide; abdomen 3.78 long, 2.03 wide. Eyes sizes and interdistances: AME 0.11, ALE 0.15, PME 0.12, PLE 0.14; AME–AME 0.12, AME–ALE 0.08, PME–PME 0.28, PME–PLE 0.19, ALE–PLE 0.11. MOA 0.40 long, front width 0.35, back width 0.52. Clypeus height 0.07. Labium 0.48 long, 0.34 wide. Sternum 1.52 long, 1.07 wide. Leg measurements: I 6.57 (1.90, 0.98, 1.73, 1.25, 0.71), II 7.33 (2.10, 1.00, 1.98, 1.50, 0.75), III 6.18 (1.84, 0.83, 1.35, 1.56, 0.60), IV 8.41 (2.32, 0.99, 1.95, 2.35, 0.80). Leg formula: 4-2-1-3. Coloration slightly lighter than in male. Other characters as in male.

Epigyne (Figs 3B, C, 5A, B). Epigynal plate as long as wide, obviously sclerotised. Copulatory openings close to each other, located almost centrally. Copulatory ducts curved, connected with bursae at anterior part of epigyne. Bursae pear-shaped, close to each other, 1.6× longer than wide. Spermathecae long, tubular, located on dorsal-inner surface of bursae. Fertilisation ducts thin and short.

#### Distribution.

Presently known only from Yunnan, China.

### ﻿ Clubiona subhuiming sp. nov.

Taxon classificationAnimaliaAraneaeClubionidae

60F42C7A-EC88-5E4E-BFD5-B54944F8FF8F

https://zoobank.org/529B6482-C138-4A26-BA5F-61CDB136F2D2

Figs 6 , 7

#### Type material.

***Holotype***: China • ♂; Hunan Province, Sangzhi County, Bamaoxi Town, Xiaozhuangping Village (29°47'30''N, 110°05'15''E, 1582 m elev.), 3 November 2018, Yang Chen leg. ***Paratypes***: • 1♀, same data as holotype.

#### Etymology.

The specific name is the combination of the prefix *sub*- (near) and *huiming*, referring to its similarity to *C.huiming* Wang, Zhang & Zhang, 2018.

#### Diagnosis.

The male of the new species is similar to *C.huiming* (Wang et al. 2018: figs 8, 9), but differs from the latter by the presence of 2 patellar apophyses (vs. 0), the smaller conductor width/genital bulb width ratio (0.4 vs. 0.7). The female of the new species is similar to *C.subapplanata* Wang, Zhang & Zhang, 2018 (Wang et al. 2018: figs 14, 15), but differs from the latter by having 2 separate copulatory openings (vs. conjoined) and coiled copulatory ducts (vs. s-shaped).

#### Description.

**Figure 6.**
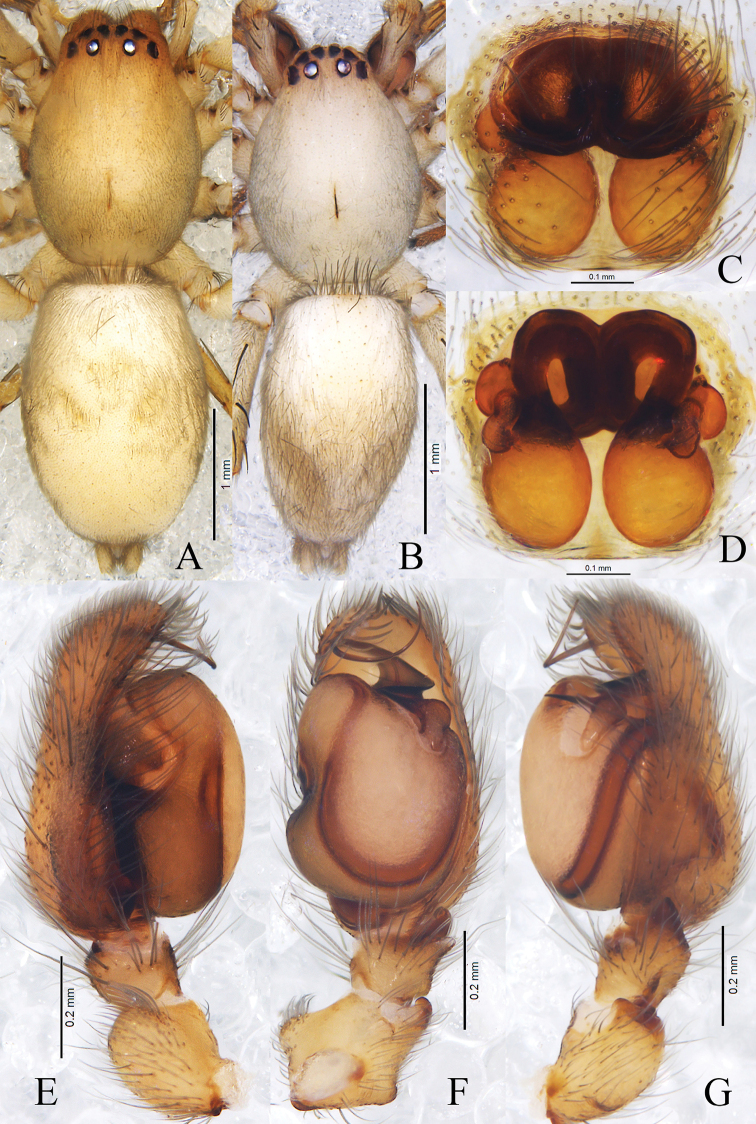
*Clubionasubhuiming* sp. nov. **A** female habitus **B** male habitus **C** epigyne, ventral view **D** vulva, dorsal view. Left male palp: **E** prolateral view **F** ventral view **G** retrolateral view. Scale bars: 1 mm (**A, B**); 0.1 mm (**C, D**); 0.2 mm (**E–G**).

**Male** (Fig. 6B). Holotype total length 3.31. Carapace 1.59 long, 1.18 wide; abdomen 1.83 long, 1.04 wide. Carapace pale yellow. Median furrow longitudinal, slightly elevated above on the carapace. In dorsal view, both anterior eye row and posterior eye row recurved. Eye sizes and interdistances: AME 0.08, ALE 0.10, PME 0.09, PLE 0.10; AME–AME 0.06, AME–ALE 0.03, PME–PME 0.14, PME–PLE 0.10, ALE–PLE 0.05. MOA 0.22 long, front width 0.20, back width 0.32. Clypeus height 0.05. Chelicerae yellowish, promargin with 5 teeth, retromargin with 4 teeth. Endites yellow, longer than wide. Labium yellow-brown, 0.20 long, 0.20 wide. Sternum 0.93 long, 0.63 wide. Abdomen oval, pale yellow, with conspicuous anterior tufts of setae; abdomen dorsum with fine, yellow hairs; venter yellow. Spinnerets and legs yellowish brown. Leg measurements: I 3.93 (1.19, 0.49, 1.06, 0.77, 0.42), II 4.26 (1.29, 0.50, 1.20, 0.81, 0.46), III 3.66 (1.07, 0.34, 0.96, 0.93, 0.36), IV 5.14 (1.47, 0.54, 1.20, 1.48, 0.45). Leg formula: 4-2-1-3.

**Figure 7.**
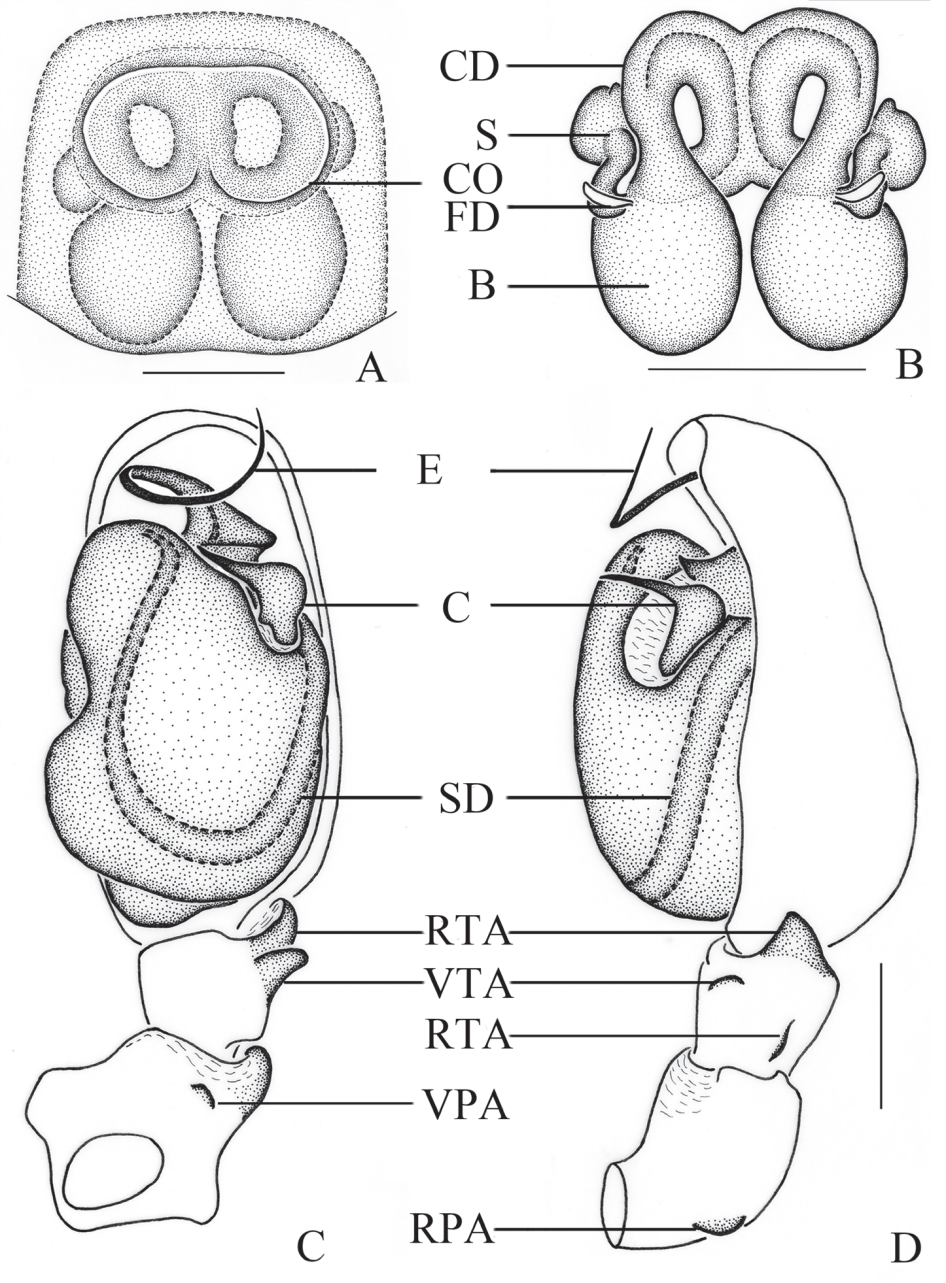
*Clubionasubhuiming* sp. nov. **A** epigyne, ventral view **B** vulva, dorsal view. Left male palp: **C** ventral view **D** retrolateral view. Scale bars: 0.25 mm (**A–D**).

Palp (Figs 6E–G, 7C, D). Patella length/width ratio 1.1, with 2 small, wide apophyses; RPA almost as long as VPA. Patella length/tibia length ratio 2.1. Tibia length/width ratio 1.4, with three apophyses, distal RTA almost conical in retrolateral view; basic RTA short, wide, and located retrolaterally; VTA thumb-shaped in ventral view, almost as long as distal RTA. Cymbium length/tibia length ratio 3.0. Cymbium length/width ratio 1.8. Genital bulb inflated, with a depression at about the 9 o’clock position. Embolus long and coiled, originated at about the 12 o’clock position, then circled clockwise, and terminated at about the 1 o’clock position. Conductor V-shaped in retrolateral view, wide in mid-part, tapering toward its apex. Sperm duct obvious, U-shaped in ventral view.

**Female** (Fig. 6A). Total length 4.04. Carapace 1.85 long, 1.33 wide; abdomen 2.16 long, 1.41 wide. Eyes sizes and interdistances: AME 0.06, ALE 0.11, PME 0.09, PLE 0.10; AME–AME 0.10, AME–ALE 0.06, PME–PME 0.20, PME–PLE 0.13, ALE–PLE 0.10. MOA 0.24 long, front width 0.24, back width 0.39. Clypeus height 0.05. Labium 0.29 long, 0.27 wide. Sternum 1.07 long, 0.72 wide. Leg measurements: I 3.71 (1.11, 0.54, 0.97, 0.66, 0.43), II 4.01 (1.18, 0.59, 1.06, 0.71, 0.47), III 3.33 (0.87, 0.47, 0.79, 0.86, 0.34), IV 5.20 (1.47, 0.61, 1.19, 1.36, 0.57). Leg formula: 4-2-1-3. Coloration darker than in male. Other characters as in male.

Epigyne (Figs 6C, D, 7A, B). Epigynal plate as long as wide, with strong sclerotised anterior part. Copulatory openings separated from each other, and meso-laterally located. Copulatory duct coiled, almost as long as bursa perimeter. Spermathecae duct-shaped, located on ventral-lateral surface of bursae. Bursae almost spherical, situated posteriorly. Fertilisation ducts lance-shaped, membranous, located on dorsal-anterior surface of bursae.

#### Distribution.

Presently known only from Hunan, China.

## ﻿Discussion

To date, nearly one-third of *Clubiona* species in China belong to *Clubionacorticalis* species group, and more than half of them have been reported for the first time in the last five years. It is likely that this species group is not monotypic, although we follow previous studies (Mikhailov 1995; Deeleman-Reinhold 2001; Zhang et al. 2021) and temporarily place these three new species in the *Clubionacorticalis* species group. More studies, therefore, are needed to further investigate the species diversity and morphological characteristics of this group of species.

## Supplementary Material

XML Treatment for Clubiona bidactylina

XML Treatment for Clubiona camela

XML Treatment for Clubiona subhuiming
